# Comparison of Sensory-Adapted and Regular Dental Environments During Professional Oral Hygiene in Children with Mild Intellectual Disability and Moderate Dental Anxiety: A Randomized Clinical Trial

**DOI:** 10.3390/medicina62081496

**Published:** 2026-08-04

**Authors:** Antonio Fallea, Simona L’Episcopo, Massimiliano Bartolone, Mirella Vinci, Luigi Vetri, Maurizio Elia, Raffaele Ferri, Simone Treccarichi, Francesco Calì

**Affiliations:** 1 Oasi Research Institute—IRCCS, 94018 Troina, Italy; afallea@oasi.en.it (A.F.); slepiscopo@oasi.en.it (S.L.); mbartolone@oasi.en.it (M.B.); mvinci@oasi.en.it (M.V.); melia@oasi.en.it (M.E.); rferri@oasi.en.it (R.F.); cali@oasi.en.it (F.C.); 2 Child Neuropsychiatry Unit of Nicosia, UOC NPIA-ASP Enna, 94014 Nicosia, Italy; luigi.vetri@asp.enna.it; 3 Department of Medicine and Surgery, Kore University of Enna, 94100 Enna, Italy

**Keywords:** intellectual disability, dental anxiety, sensory-adapted dental environment, pediatric dentistry, FLACC, Frankl scale, pain perception, patient cooperation

## Abstract

*Background and Objectives*: Children with mild intellectual disability (MID) frequently experience difficulties during dental treatment due to increased sensitivity to environmental stimuli and limited cooperation. Sensory-adapted dental environments (SADE) have been proposed as a strategy to improve the dental experience of patients with neurodevelopmental conditions; however, evidence regarding their effectiveness in children with MID remains limited. This randomized clinical trial aimed to evaluate the effects of a sensory-adapted dental environment on pain-related behaviors and treatment cooperation during professional oral hygiene procedures in children with MID and moderate dental anxiety. *Materials and Methods*: A randomized clinical trial was conducted at the IRCCS Oasi Research Institute (Troina, Italy). A total of 200 children were screened for eligibility. Following the application of the predefined inclusion and exclusion criteria, 160 children (76 males and 84 females; age range: 6–7 years) with a diagnosis of mild intellectual disability (MID) and moderate dental anxiety, assessed using the Corah Dental Anxiety Scale (DAS), were enrolled. Participants were randomly assigned to either a sensory-adapted dental environment (SADE; n = 80) or a regular dental environment (RDE; n = 80). All participants underwent a single session of professional oral hygiene using an ultrasonic scaler. Pain-related behaviors were assessed using the Face, Legs, Activity, Cry, Consolability (FLACC) Scale, whereas cooperation during treatment was evaluated using the Frankl Behavior Rating Scale. Owing to the non-normal distribution of the outcome variables, between-group comparisons were performed using the Mann–Whitney U test. Statistical significance was set at *p* < 0.05. *Results*: Participants treated in the SADE showed significantly lower FLACC scores compared with those treated in the RDE (3.10 ± 1.86 vs. 5.05 ± 2.36, *p* < 0.001), indicating reduced pain-related behaviors during treatment. Moreover, children in the SADE group demonstrated significantly higher Frankl scores than those in the RDE group (2.78 ± 0.48 vs. 2.41 ± 0.84, *p* = 0.006), reflecting improved cooperation. Sex-stratified analyses confirmed significantly lower FLACC scores in both males and females treated in the SADE. No significant Group × Sex interaction was observed for either outcome, suggesting that the beneficial effects of sensory adaptation were independent of sex. *Conclusions*: SADE significantly reduced pain-related behaviors and improved cooperation during professional oral hygiene procedures in children with mild intellectual disability and moderate dental anxiety. These findings support the implementation of sensory-adapted strategies in pediatric dental settings to improve treatment experiences and facilitate dental care for children with neurodevelopmental disorders.

## 1. Introduction

Children with NDDs frequently experience significant challenges during dental treatment. Cognitive impairments, communication difficulties, sensory sensitivities, and heightened anxiety may contribute to reduced cooperation and increased distress during oral healthcare procedures [1]. Consequently, individuals with NDDs often experience poorer oral health outcomes and face both physical and behavioral barriers to accessing routine dental care compared with their typically developing peers [2]. The implementation of innovative behavioral, technological, and patient-centered approaches has significantly improved the management of dental care in children with NDDs [3,4,5]. These strategies contribute to reducing anxiety, enhancing patient cooperation, and facilitating communication during dental procedures. Furthermore, they provide substantial benefits not only for children, by promoting a more positive and less stressful clinical experience, but also for caregivers, who often face considerable challenges in accessing oral healthcare services, and for dental professionals, who can perform examinations and treatments more effectively and safely. Based on these considerations, children presenting with NDDs often require specialized dental care tailored to their individual needs [6,7]. Cognitive, behavioral, communicative, and sensory challenges may interfere with the delivery of routine oral healthcare, making standard dental approaches less effective. Consequently, dental treatment frequently needs to be individualized and adapted according to each patient’s specific functional profile and support requirements.

To address these challenges, sensory-adapted dental environments (SADEs) have been developed as non-pharmacological interventions aimed at reducing sensory overstimulation and improving the dental experience [8,9]. SADEs typically incorporate environmental modifications such as dimmed lighting, audiovisual distraction, noise reduction strategies, and other sensory accommodations designed to create a more comfortable and less stressful clinical setting. Previous studies have demonstrated that sensory adaptation may reduce anxiety-related behaviors, improve cooperation, and enhance treatment compliance, particularly among individuals with autism spectrum disorder and other neurodevelopmental conditions [8,10]. The concept of a sensory-adapted dental environment is grounded in sensory integration theory, which recognizes that atypical sensory processing may contribute to heightened anxiety, behavioral dysregulation, and avoidance of healthcare settings among individuals with neurodevelopmental conditions [11,12]. Rather than relying on a single intervention, SADE represents a multicomponent approach that systematically modifies environmental stimuli—including visual, auditory, tactile, and, when appropriate, proprioceptive input—to reduce sensory overload while maintaining the quality and safety of dental care [12,13]. Typical adaptations include reduced ambient lighting, minimization of unexpected sounds, calming audiovisual projections or music, weighted blankets or deep-pressure supports, simplified operatory organization, and structured communication strategies. The selection and combination of these adaptations should be individualized according to each patient’s sensory profile and clinical needs, with the overall objective of promoting self-regulation, reducing distress, and facilitating successful completion of dental procedures [13].

Despite these promising findings, evidence regarding the effectiveness of sensory-adapted dental environments in children with mild intellectual disability remains limited. Given the unique cognitive and behavioral characteristics of this population, further investigation is warranted to determine whether sensory adaptation can improve cooperation and reduce treatment-related discomfort during routine dental procedures.

Therefore, the aim of the present randomized clinical trial was to compare SADE with RDE during professional oral hygiene procedures in children with mild intellectual disability and moderate dental anxiety.

## 2. Materials and Methods

### 2.1. Patient Enrollment

A randomized clinical trial was conducted at the IRCCS Oasi Research Institute (Troina, Enna, Italy). The study was registered at ClinicalTrials.gov (Identifier: NCT07413016) prior to participant recruitment, with registration completed on 9 February 2026. Patient enrollment began in February 2026, and the primary completion date was May 2026. The study protocol was approved by the local Ethics Committee (CEL-IRCCS Oasi Maria SS.; Protocol No. CEL-IRCCS OASI/10-04-2025/02). This study adheres to the CONSORT guidelines. Written informed consent was obtained from the parents or legal caregivers of all participants. All procedures were conducted in accordance with the ethical principles of the Declaration of Helsinki.

At enrollment, all participants underwent assessment of dental anxiety using the Corah Dental Anxiety Scale (DAS). During the recruitment period, all consecutive children aged 6–7 years with a confirmed diagnosis of mild intellectual disability who attended the IRCCS Oasi Research Institute for professional oral hygiene were screened for eligibility. All children with MID attending the clinic during the recruitment period were screened, and no potentially eligible patients were missed.

### 2.2. Inclusion and Exclusion Criteria

Participants were eligible for inclusion if they met the following criteria: (i) age between 6 and 7 years; (ii) a confirmed diagnosis of mild intellectual disability (MID) established by neuropsychiatrists according to DSM-5 criteria, integrating standardized measures of intellectual functioning with clinical assessment of adaptive functioning. Although participants presented intellectual functioning within the range historically associated with mild intellectual disability (approximately IQ 50–70), the diagnosis and severity classification were based primarily on adaptive functioning, in accordance with DSM-5; and (iii) moderate dental anxiety as assessed by the DAS [14,15]. This scale consisted of four-item questionnaire designed to assess anxiety associated with common dental situations. Each item is rated on a five-point Likert scale, yielding a total score ranging from 4 to 20. Anxiety levels are classified as follows: no anxiety (score = 4), slight anxiety (scores 5–8), moderate anxiety (scores 9–12), high anxiety (scores 13–14), and severe anxiety (scores 15–20). Given the cognitive and communicative characteristics of children with MID, the DAS was administered through a structured interview rather than self-completion. This approach allowed the examiner to clarify questions, adapt the pace of administration, and facilitate accurate reporting of anxiety levels. To ensure consistency, all interviews were conducted by the same trained examiner following standardized administration and scoring procedures. DAS assessment was performed during a preliminary evaluation by an examiner who was blinded to the study hypotheses and experimental conditions.

Exclusion criteria included the absence of anxiety (DAS score = 4), slight anxiety (DAS scores 5–8), high anxiety (DAS scores 13–14), severe anxiety (DAS scores 15–20), visual impairments, and neuromuscular disorders that could interfere with cooperation during the study procedures.

**Figure 1:**
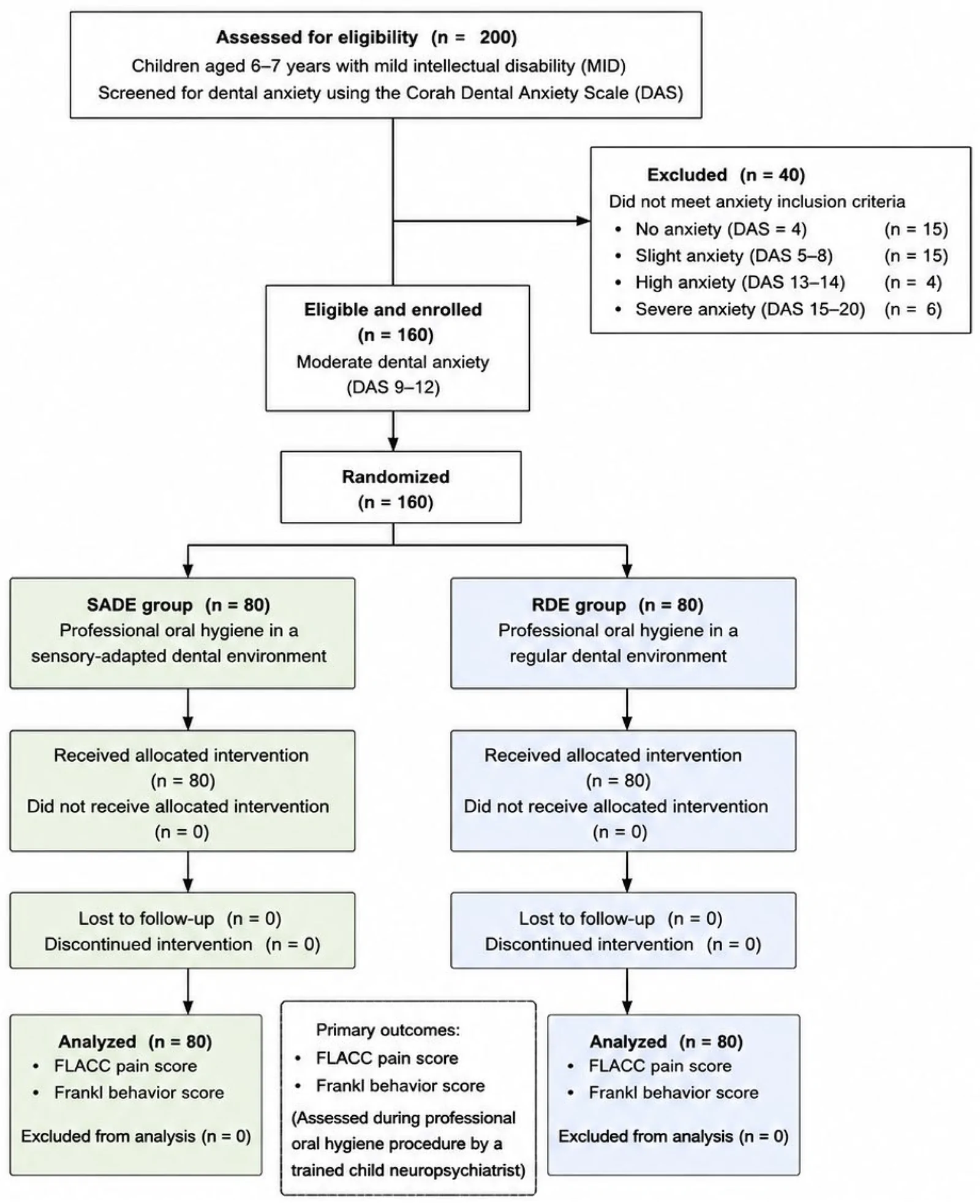
Flow diagram illustrating participant enrollment, eligibility assessment, allocation to the sensory-adapted dental environment (SADE) or regular dental environment (RDE) groups, follow-up, and final analysis.

A total of 200 children were screened for eligibility. All 200 children screened for eligibility had a confirmed diagnosis of mild intellectual disability according to DSM-5 criteria. Of these, 40 were excluded because they did not meet the anxiety-related inclusion criteria established for the study. According to DAS assessment, 15 children exhibited no anxiety, 15 slight anxiety, 4 high anxiety, and 6 severe anxiety. Therefore, 160 participants were included in the study and underwent randomization. All participants met the criteria for moderate dental anxiety, defined as a DAS score between 9 and 12. The final study population comprised 76 males and 84 females. In addition, only children requiring professional oral hygiene because of the presence of dental plaque and calculus identified during the baseline dental examination were considered eligible for enrollment. Figure 1 illustrates the flow diagram of this clinical trial.

### 2.3. Randomization and Intervention

After enrollment, participants were randomly allocated to one of two study groups using a balanced sex distribution:

- SADE group (n = 80): participants underwent a professional oral hygiene session in a modified dental environment.
- RDE control group (n = 80): participants underwent a professional oral hygiene session in a standard, non-modified dental environment.

After enrollment, participants were stratified by sex and randomly assigned to the SADE or RDE group in a 1:1 allocation ratio using a computer-generated random sequence. Randomization was performed separately within each sex stratum to promote comparability in sex distribution between the two groups. The procedure resulted in two groups of equal overall size (n = 80 each), with no statistically significant difference in sex distribution between groups.

During all professional oral hygiene procedures, pain-related behaviors and treatment cooperation were assessed by a trained child neuropsychiatrist who was not involved in the dental treatment. Owing to the evident environmental differences between the SADE and RDE settings, the assessor could not be blinded to group allocation. Pain perception was assessed using the Face, Legs, Activity, Cry, Consolability (FLACC) Scale, whereas patient cooperation and behavior during treatment were assessed using the Frankl Behavior Rating Scale.

### 2.4. FLACC Scale

The FLACC scale is an observational tool commonly used to assess pain in children who are unable to communicate effectively or who have cognitive impairments [16,17]. The scale evaluates five behavioral domains (face, legs, activity, cry, and consolability), each scored from 0 to 2. The total score ranges from 0 to 10, with higher scores indicating greater pain intensity.

### 2.5. Frankl Behavior Rating Scale

The Frankl Behavior Rating Scale is a widely used instrument for evaluating children’s behavior and cooperation during dental treatment [18,19]. The scale classifies patient behavior into four categories: definitely negative (score 1), negative (score 2), positive (score 3), and definitely positive (score 4). Higher scores indicate greater cooperation and a more favorable attitude toward dental procedures.

### 2.6. Professional Oral Hygiene Procedure

All participants received a single session of professional oral hygiene treatment performed in a dental chair by the same special care dentist with more than 20 years of clinical experience in special care dentistry, including the management of patients with neurodevelopmental disorders. The professional oral hygiene treatment lasted approximately 30 min. Supragingival plaque and calculus removal were carried out using an ultrasonic scaler according to standard clinical procedures.

### 2.7. SADE

Participants allocated to the SADE group received treatment in a modified dental environment specifically designed to reduce the impact of sensory stimuli potentially associated with anxiety and discomfort during dental treatment. The clinical setting was adapted according to principles aimed at minimizing visual and auditory stimulation while maintaining appropriate standards of clinical safety and operational efficiency.

**Figure 2:**
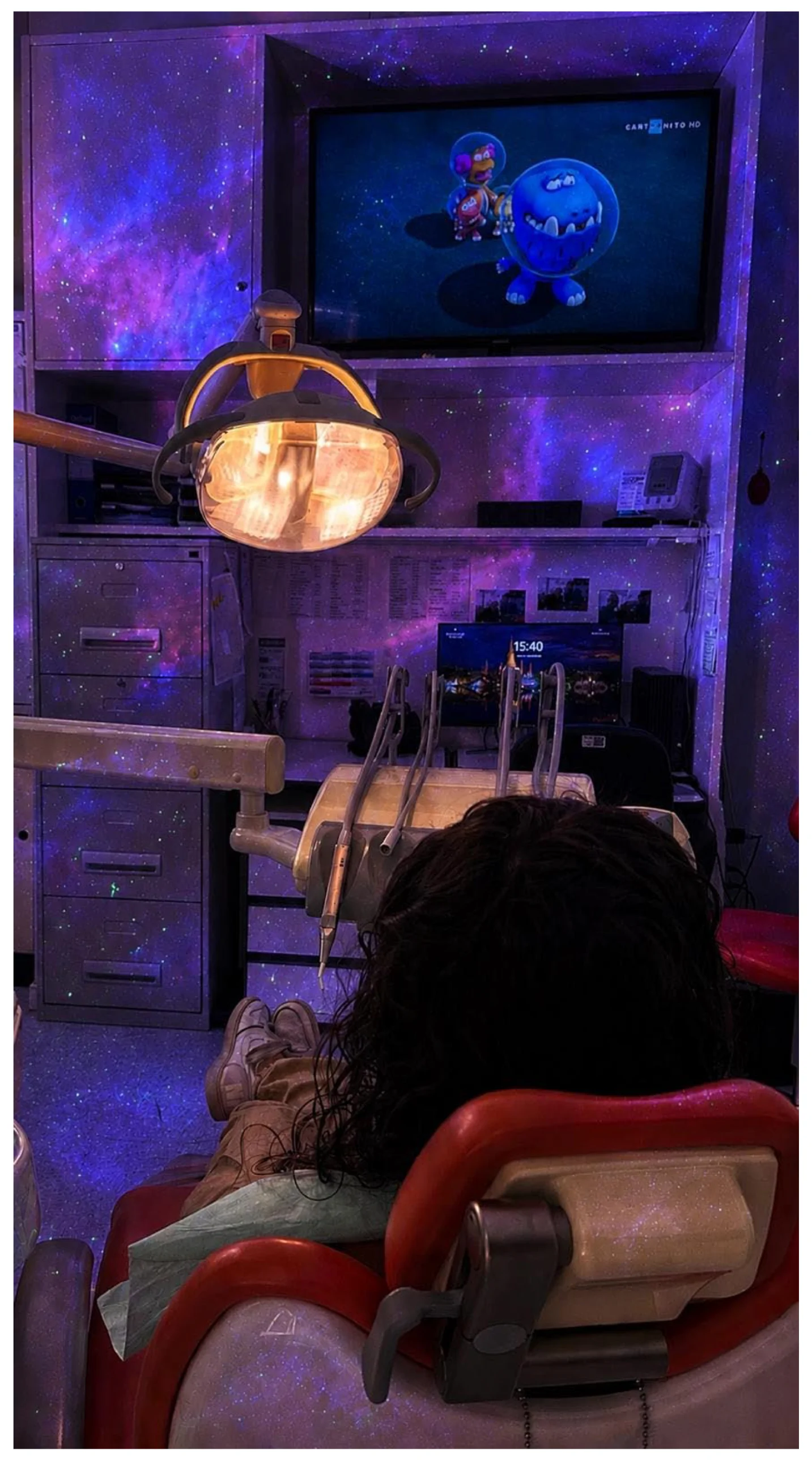
Sensory-adapted dental environment (SADE) used during professional oral hygiene procedures. The clinical setting was modified to minimize sensory over-stimulation by implementing multiple environmental adaptations, including dimmed ambient lighting, calming galaxy-like visual projections on the walls and ceiling, and audiovisual distraction delivered through a ceiling-mounted monitor displaying age-appropriate animated content. The dental operating light remained focused exclusively on the treatment field to preserve clinical visibility while minimizing unnecessary visual stimulation. These sensory accommodations were designed to create a calming clinical atmosphere, to enhance patient comfort and reduce treatment-related anxiety, and facilitate cooperation during oral hygiene procedures in children with mild intellectual disability.

Environmental modifications included reduced-intensity ambient lighting intended to create a more relaxing atmosphere compared with conventional dental clinics. During dental procedures, the illumination required for the operative field was maintained according to clinical needs, whereas the surrounding ambient lighting remained attenuated. To facilitate distraction and reduce attention toward the dental procedure, a large monitor was positioned to ensure optimal visibility from the dental chair. The screen displayed audiovisual entertainment content, including cartoons, movies, and other videos selected according to the child’s age and personal preferences. Whenever possible, audiovisual materials were chosen in advance by the family to maximize familiarity and engagement. To further reduce sensory discomfort, procedures were performed using a sponge-coated, low-noise dental turbine handpiece specifically designed to minimize acoustic emissions commonly associated with dental treatment. This modification substantially reduced the noise perceived during operative procedures, thereby contributing to a more comfortable and less stressful treatment environment. Figure 2 shows the sensory-adapted dental environment implemented for participants allocated to the SADE group.

### 2.8. RDE

Participants allocated to the RDE control group received treatment in a conventional dental operatory without environmental modifications. The room maintained the standard clinical setting routinely used for pediatric dental procedures at the IRCCS Oasi Research Institute.

### 2.9. Data Analysis

Statistical analyses were performed using R software (version 4.5.2.). Descriptive statistics were calculated for all study variables. Continuous and ordinal variables were reported as mean ± standard deviation and median with interquartile range, as appropriate. Categorical variables were summarized as frequencies and percentages. The normality of FLACC and Frankl scale scores was assessed using the Shapiro–Wilk test. Since these variables were ordinal and/or not normally distributed, comparisons between the SADE and RDE groups were performed using the Mann–Whitney U test. The distribution of sex between groups was evaluated using the chi-square test or Fisher’s exact test, as appropriate. To explore whether treatment effects differed according to sex, additional sex-stratified analyses were performed. Specifically, FLACC and Frankl scores were compared between the SADE and RDE groups separately in males and females using the Mann–Whitney U test. Where multiple stratified comparisons were performed, *p*-values were adjusted using the Bonferroni correction. In addition, interaction analyses were conducted to assess the potential Group × Sex effect on FLACC and Frankl scores. A two-sided *p*-value < 0.05 was considered statistically significant. In addition to *p*-values and effect sizes (r), 95% confidence intervals were calculated where appropriate. For FLACC scores, the Hodges–Lehmann estimator with its 95% confidence interval was reported to quantify the between-group difference. For Frankl scores, owing to the ordinal nature of the scale and the high frequency of tied observations, the rank-biserial correlation with its 95% confidence interval was calculated.

Post hoc power analysis was performed using G*Power software (version 3.1; Heinrich Heine University Düsseldorf, Germany) based on the primary outcome (FLACC score). Using the observed standardized effect size (Cohen’s *d* = 0.92), a two-sided significance level of 0.05, and an allocation ratio of 1:1, the required sample size was estimated at 20 participants per group to achieve 80% statistical power and 26 participants per group to achieve 90% statistical power. Therefore, the final sample of 80 participants per group provided an estimated statistical power greater than 99% for detecting the observed between-group difference.

## 3. Results

Of the 200 children assessed for eligibility, 160 (80.0%) met the predefined inclusion criteria and were enrolled in the study, whereas 40 (20.0%) were excluded because they did not meet the dental anxiety eligibility criteria. Specifically, 15 children (7.5%) had no dental anxiety (DAS = 4), 15 (7.5%) had slight dental anxiety (DAS 5–8), 4 (2.0%) had high dental anxiety (DAS 13–14), and 6 (3.0%) had severe dental anxiety (DAS 15–20). A total of 160 children were randomized to either the SADE group (n = 80) or the RDE group (n = 80). The distribution of sex did not differ significantly between groups (χ^2^ = 0.63, *p* = 0.426), confirming adequate balance after randomization. Baseline demographic and clinical characteristics of the participants are summarized in Table 1.

The Shapiro–Wilk test revealed that both FLACC and Frankl scores were not normally distributed in either study group (all *p* < 0.01) (Table 2).

Therefore, non-parametric analyses were performed. Patients treated in the sensory-adapted dental environment (SADE) showed significantly lower FLACC scores than those treated in the regular dental environment (RDE) (mean ± SD: 3.10 ± 1.86 vs. 5.05 ± 2.36; Mann–Whitney U test, *p* < 0.001), with a moderate effect size (r = 0.417). he Hodges–Lehmann estimate indicated a 2-point reduction in FLACC scores in the SADE group compared with the RDE group (estimate: −2; 95% CI: −3 to −1). Similarly, Frankl scores were significantly higher in the SADE group than in the RDE group (mean ± SD: 2.78 ± 0.48 vs. 2.41 ± 0.84; Mann–Whitney U test, *p* = 0.006), indicating better behavioral cooperation during treatment, although the effect size was small (r = 0.217). The rank-biserial correlation was 0.198 (95% CI: 0.061 to 0.332), indicating a small effect favoring the SADE group. To evaluate whether sex modified the treatment effect, interaction analyses were performed using a rank-based non-parametric model including Group, Sex, and the Group × Sex interaction term. No significant Group × Sex interaction was observed for either FLACC scores (*p* = 0.271) or Frankl scores (*p* = 0.748), indicating that the beneficial effects of the sensory-adapted dental environment were comparable between males and females.

## 4. Discussion

The present randomized clinical trial investigated the effects of SADE on pain-related behaviors and treatment cooperation during professional oral hygiene procedures in children with MID and moderate dental anxiety. A total of 160 participants were randomly assigned to either a sensory-adapted dental environment or a RDE. The main findings demonstrated that children treated in the SADE exhibited significantly lower FLACC scores, indicating reduced pain-related behaviors, and significantly higher Frankl scores, reflecting improved behavioral cooperation during treatment. These results suggest that sensory adaptation of the dental setting may effectively reduce treatment-related distress and facilitate patient compliance during routine oral hygiene procedures. The observed benefits may be explained by the reduction in sensory overstimulation frequently experienced by children with neurodevelopmental disorders in conventional dental settings. Dental clinics are characterized by multiple sensory stimuli, including bright lights, unfamiliar sounds, tactile sensations, and odors, which may trigger discomfort, anxiety-related responses, and behavioral dysregulation. By modifying these environmental characteristics, sensory-adapted settings may promote emotional regulation, reduce sensory burden, and facilitate a more positive treatment experience. Consequently, children may be more willing to cooperate with dental procedures and exhibit fewer pain-related behavioral responses. The present findings are consistent with previous studies reporting beneficial effects of sensory-adapted dental environments in children with neurodevelopmental disorders, particularly ASD [9,20,21]. Previous investigations have demonstrated reductions in physiological and behavioral indicators of distress, supporting the hypothesis that sensory modifications can positively influence the dental experience. Although physiological measures were not collected in the present study, the FLACC Scale and the Frankl Behavior Rating Scale provided clinically meaningful indicators of pain-related behaviors and treatment cooperation, respectively [22,23,24]. Together, these findings further support the utility of sensory-adapted approaches in improving the delivery of oral healthcare to children with developmental disabilities.

An important finding of the present study is that the beneficial effects of sensory adaptation were independent of sex. No significant interaction between treatment group and sex was observed for either FLACC or Frankl scores, indicating that the positive effects of the sensory-adapted environment were comparable in males and females. This observation is particularly noteworthy because many previous studies involving neurodevelopmental disorders, especially ASD, have been characterized by a marked predominance of male participants, reflecting the higher prevalence of these conditions among males [9]. A preliminary study investigating the effects of sensory-adapted dental environments was previously conducted in a relatively balanced cohort of individuals with intellectual disability; however, the study population consisted of adults rather than children [25].

Consequently, evidence regarding potential sex-related differences in response to sensory-adapted interventions remains limited. The relatively balanced male-to-female ratio of the present cohort allowed a more robust evaluation of this aspect and provides preliminary evidence that sensory adaptation may be equally effective across sexes in children with mild intellectual disability.

From a clinical perspective, the findings of the present study highlight the practical value of sensory-adapted environments in pediatric dentistry. Improved cooperation during treatment may facilitate the completion of preventive and therapeutic procedures while reducing the need for more invasive behavior management strategies. In this context, the Frankl Behavior Rating Scale represents a particularly useful instrument for assessing treatment cooperation in children with neurodevelopmental disorders [9,21]. Unlike physiological measurements, which may require additional equipment and can be difficult to obtain in children with limited compliance, the Frankl Scale is non-invasive, easy to administer, and readily applicable during routine clinical practice. These characteristics make it a valuable and ecologically valid tool for evaluating behavioral responses during dental treatment.

It should be emphasized that the primary aim of the present study was not to evaluate the reduction in dental anxiety itself. Moderate dental anxiety, as assessed by the Corah Dental Anxiety Scale, was used exclusively as an inclusion criterion to ensure a homogeneous study population. Since anxiety levels were not reassessed following treatment, no conclusions can be drawn regarding a direct effect of the sensory-adapted environment on dental anxiety per se. Nevertheless, the significantly lower FLACC scores and higher Frankl scores observed in the SADE group suggest that sensory adaptation may attenuate the behavioral and psychophysiological manifestations commonly associated with dental anxiety during treatment. Therefore, the benefits observed in the present study should be interpreted primarily as improvements in pain-related behaviors and treatment cooperation rather than as direct evidence of anxiety reduction.

Future studies should further explore the mechanisms underlying the beneficial effects of sensory-adapted dental environments by integrating objective physiological and behavioral measures. Parameters such as heart rate variability, electrodermal activity, oxygen saturation, and salivary biomarkers may provide additional information regarding stress, autonomic activation, and nociceptive responses during dental treatment. Furthermore, the implementation of audio- and video-recording systems combined with machine learning and artificial intelligence approaches may represent a promising strategy for objectively assessing cooperation, discomfort, and emotional responses. Automated analysis of facial expressions, body movements, vocalizations, and other behavioral indicators could complement conventional clinical scales, including the FLACC and Frankl scales, by providing continuous, observer-independent, and quantitatively measurable data. Such approaches may ultimately contribute to the development of more precise and personalized behavioral assessment tools for children with neurodevelopmental disorders.

Overall, the results of the present study support the incorporation of sensory-adapted approaches into pediatric dental practice for children with mild intellectual disability and elevated levels of dental anxiety. By reducing pain-related behaviors and improving cooperation during treatment, sensory-adapted environments may facilitate access to oral healthcare and contribute to a more positive dental experience for both patients and healthcare providers.

Several limitations should be considered when interpreting the findings of the present study. First, objective physiological measures of stress and pain, such as oxygen saturation, heart rate variability, electrodermal activity, and salivary biomarkers, were not collected. The inclusion of these parameters could have provided a more comprehensive assessment of autonomic and nociceptive responses associated with dental treatment [26]. Moreover, physiological and biochemical markers may have complemented the behavioral measures used in this study, allowing a more objective characterization of treatment-related stress and discomfort.

A second limitation concerns the impossibility of blinding the dental hygienist performing the oral hygiene procedures. Because the operator was aware of the treatment allocation (SADE or RDE), a certain degree of performance bias cannot be completely excluded. Although all procedures were conducted according to a standardized protocol by the same experienced hygienist, subtle differences in operator–patient interaction may have influenced patient behavior and cooperation. Nevertheless, the use of a single operator ensured procedural consistency across participants and minimized inter-operator variability. Another limitation of the present study is the potential observer bias arising from the impossibility of blinding the outcome assessor. Because the sensory-adapted dental environment was characterized by evident environmental modifications, including dimmed lighting, visual projections, and audiovisual distraction, blinding to group allocation was not feasible. This may have introduced the possibility of detection bias when assessing behavioral outcomes. Nevertheless, to minimize this potential source of bias, all assessments were performed by the same experienced child neuropsychiatrist using two standardized and validated behavioral scales (FLACC and Frankl), following predefined scoring criteria. Furthermore, the consistency of the findings across both outcome measures strengthens the reliability of the observed treatment effect.

## 5. Conclusions

Sensory-adapted dental environments represent an effective behavioral strategy for improving preventive oral healthcare in children with mild intellectual disability. From a clinical perspective, the implementation of sensory-adapted dental environments may improve the delivery of preventive dental care by enhancing patient cooperation and reducing pain-related behaviors.

## Figures and Tables

**Table 1:** Baseline demographic and clinical characteristics of the study participants.

Characteristic	RDE (n = 80)	SADE (n = 80)	*p*-Value
Age (years), mean ± SD	6.46 ± (0.5)	6.54 ± (0.5)	0.345
Male/Female, n	32/48	38/42	0.426
DAS, mean ± SD	10.44 ± 1.43	10.46 ± 1.12	0.554

**Table 2:** Comparison of FLACC and Frankl scores between study groups. Values are presented as mean ± standard deviation (SD) and median (interquartile range, IQR). *p*-values were obtained using the Mann–Whitney U test.

Variable	RDE	SADE	*p*-Value	Effect Size (r)	Effect Estimate (95% CI) *
FLACC, mean ± SD	5.05 ± 2.36	3.10 ± 1.86	<0.001	0.417	HL estimate −2 (95% CI: −3 to −1)
FLACC, median (IQR)	5 (2)	3 (2)			
Frankl, mean ± SD	2.41 ± 0.84	2.78 ± 0.48	0.006	0.217	Rank-biserial correlation: 0.198 (95% CI: 0.061 to 0.332)
Frankl, median (IQR)	2 (1)	3 (0)			

* For FLACC, the estimate represents the Hodges–Lehmann estimator of the between-group difference. For Frankl, the estimate represents the rank-biserial correlation with its 95% confidence interval, owing to the ordinal nature of the outcome.

## Data Availability

The original contributions presented in this study are included in the article. Further inquiries can be directed to the corresponding author.

## Abbreviations

The following abbreviations are used in this manuscript:

ASD	Autism spectrum disorder
DAS	Dental anxiety scale
FLACC	Face, Legs, Activity, Cry, Consolability
MID	Mild intellectual disability
NDD	Neurodevelopmental disorders
RDE	Regular dental environment
SADE	Sensory-adapted dental environments
